# Efficient Tetracycline Hydrochloride Degradation by Urchin-Like Structured MoS_2_@CoFe_2_O_4_ Derived from Steel Pickling Sludge via Peroxymonosulfate Activation

**DOI:** 10.3390/molecules30153194

**Published:** 2025-07-30

**Authors:** Jin Qi, Kai Zhu, Ming Li, Yucan Liu, Pingzhou Duan, Lihua Huang

**Affiliations:** 1 College of Resources and Environment, Linyi University, Linyi 276000, China; qijinlyu@163.com (J.Q.); lm1095047380@126.com (M.L.); 2 School of Civil Engineering, Yantai University, Yantai 264005, China; liuyucan@ytu.edu.cn; 3 State Key Laboratory of Environmental Criteria and Risk Assessment, Chinese Research Academy of Environmental Sciences, Beijing 100012, China; duanpz@craes.og.cn

**Keywords:** pickling sludge, MoS_2_@CoFe_2_O_4_ catalyst, peroxymonosulfate, degradation, tetracycline hydrochloride

## Abstract

Steel pickling sludge serves as a valuable iron source for synthesizing Fe-based catalysts in heterogeneous advanced oxidation processes (AOPs). Here, MoS_2_@CoFe_2_O_4_ catalyst derived from steel pickling sludge was prepared via a facile solvothermal approach and utilized to activate peroxymonosulfate (PMS) for tetracycline hydrochloride (TCH) degradation. Comprehensive characterization using scanning electron microscopy (SEM)-energy dispersive spectrometer (EDS), X-ray photoelectron spectroscopy (XPS), and X-ray diffraction (XRD) confirmed the supported microstructure, composition, and crystalline structure of the catalyst. Key operational parameters—including catalyst dosage, PMS concentration, and initial solution pH—were systematically optimized, achieving 81% degradation efficiency within 30 min. Quenching experiments and electron paramagnetic resonance (EPR) analysis revealed SO_4_^∙−^ as the primary oxidative species, while the catalyst maintained high stability and reusability across cycles. TCH degradation primarily occurs through hydroxylation, decarbonylation, ring-opening, and oxidation reactions. This study presents a cost-effective strategy for transforming steel pickling sludge into a high-performance Fe-based catalyst, demonstrating its potential for practical AOP applications.

## 1. Introduction

The increasing discharge of wastewater from industry and domestic life, which contains huge amounts of emerging pollutants, causes potential harm to ecological security and human health [1]. The conventional wastewater treatment plants possess negligible removal capacity of these pollutants owing to their stable chemical structures and biotoxicity [2].

Conventional wastewater treatment processes poorly remove emerging pollutants, necessitating appropriate treatment technology to achieve efficient degradation while avoiding harmful byproducts. Advanced oxidation processes (AOPs) are widely employed for degrading refractory organic pollutants in wastewater [3]. These processes generate reactive oxygen species (ROS), such as hydroxyl radicals (∙OH) and sulfate radicals (SO_4_^∙−^), via the activation of H_2_O_2_, peroxymonosulfate (PMS), or peroxodisulfate (PDS) [4]. Compared to ∙OH, SO_4_^∙−^ offers superior oxidation capabilities, including a longer half-life (30–40 μs), higher redox potential (2.5–3.1 V), and pH-independent reactivity [5,6]. In typical persulfate-advanced oxidation processes (PS-AOPs), SO_4_^∙−^ can be produced by the activation of PMS/PDS through direct energy input (e.g., heat, ultrasound, UV irradiation) or by catalyst containing transition-metal ions (e.g., Fe^2+^, Co^2+^, Cu^2+^) [7,8,9]. Among these methods, heterogeneous catalysts have attracted more and more attention because of their relatively low cost of usage and high recycling capacity, avoiding the secondary pollution [10].

The design and synthesis of heterogeneous catalysts include the screening of active components to satisfy the requirements and suitable synthesis methods for catalytic performance outcomes. Many researchers have studied the iron-based catalysts for their excellent catalytic activity, broad application, and magnetic properties, as the catalysts can be conveniently separated from the reaction system for reuse [11,12]. Among these catalysts, bimetallic iron-based catalysts, which are possibly one significant subject that have inward increasing interest from researchers, possess the synergetic effects due to the interaction between different metallic elements [13]. In this context, other metallic elements can advantage the Fe^3+^/Fe^2+^ redox cycle, greatly improve the efficiency of the bimetallic iron-based catalysts [14]. At present, there have been many studies proving that the representative bimetallic iron-based catalysts, such as NiFe_2_O_3_, Fe_3_O_4_@Cu, and CuFe_2_O_3_, exhibit superior catalytic performance and a more stable structure, compared with corresponding monometallic counterparts (e.g., Fe_2_O_3_ or Fe_3_O_4_) [15,16]. In these doped metals, cobalt is considered to provide outstanding catalysis toward PMS [17]. Diverse novel heterogeneous Fe-Co bimetallic catalysts have been developed and used in the PS-AOPs because of the following advantages: (i) The Co-Fe bimetallic catalysts show preeminent activation capacity for PMS and strong magnetism for the convenient solid–liquid separation. (ii) The synergetic effects between Co-Fe can greatly facilitates the Fe^3+^/Fe^2+^ redox cycle and limits the leaching of metal ions [13,14]. Therefore, it is indispensable to develop a convenient synthesis method and find enough renewable resources to prepare Fe-Co bimetallic catalysts on a large scale [18,19].

Steel pickling sludge is an unavoidable secondary product during the removal of steel rust and purification of the original steel by HCl or H_2_SO_4_. A great deal of steel pickling sludge, approximately 1% of total steel production, is generated in the purification process [20,21]. In 2019, China produced about 1 billion tons of steel and generated about 10 million tons of steel pickling sludge, which requires proper treatment and disposal [22]. However, the dominating disposal methods for steel pickling sludge are solid waste landfill, brick manufacturing, and ferric salt flocculant manufacturing [23]. For the solid waste containing wide range of Fe and other metal elements, the transformation of metal elements into environmental catalysts for the degradation of pollutants will be a high-value treatment method. Therefore, it is of great significance to study the recovery method of valuable metal elements from steel pickling slag.

In this study, magnetic urchin-like MoS_2_@CoFe_2_O_4_ catalyst was synthesized via a facile two-step solvothermal method, using steel pickling sludge as the Fe source. This approach addresses critical research gaps in industrial waste recycling: existing studies on waste-derived catalysts for AOPs have predominantly focused on single-metal utilization or simple material conversion rather than integrating such industrial waste with functional nanomaterials to enhance catalytic performance. Moreover, prior efforts to valorize steel pickling sludge rarely explored its potential in constructing high-performance bimetallic catalysts with synergistic effects. The catalyst was then applied as a PMS activator in AOPs to degrade tetracycline hydrochloride (TCH). As a broad-spectrum antibiotic, TCH is extensively used in medicine, aquaculture, and livestock industries, leading to its frequent detection in various water matrices (e.g., wastewater, surface water, and soil) due to incomplete metabolism in organisms and improper disposal. This widespread occurrence makes it a representative pollutant for evaluating the effectiveness of wastewater treatment processes, particularly AOPs targeting emerging pollutants. The catalytic performance was systematically evaluated, focusing on the influence of support structure and key operational parameters. Results demonstrated that the MoS_2_@CoFe_2_O_4_ catalyst achieved high TCH degradation efficiency, which is attributed to the synergistic effect between MoS_2_ and CoFe_2_O_4_. Furthermore, the dominant reactive oxygen species (ROS) were identified, and the mechanism was elucidated. By transforming steel pickling sludge into a high-performance catalyst through rational integration with MoS_2_, this work fills these gaps, reducing waste disposal burdens while providing low-cost solutions for pollutant degradation. This work provides a sustainable approach to designing low-cost catalysts from industrial waste for environmental remediation.

## 2. Results and Discussion

### 2.1. Comparison of Degradation Efficiencies by Different Catalysts

**Figure 1:**
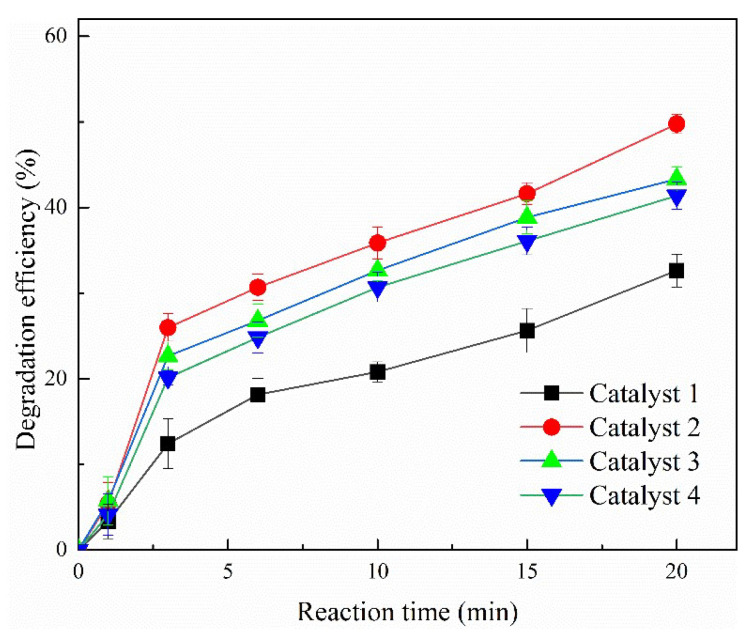
The comparison of degradation efficiencies for TCH by different catalysts (TCH 200 mg/L, PMS 300 mg/L, catalyst dosage 150 mg/L, and initial solution pH 3.5).

For the comparison of the PMS activation capacities by the prepared catalysts, catalyst 1, catalyst 2, catalyst 3, and catalyst 4 were added into TCH solutions under the condition of initial solution pH value of 3.5, catalyst concentration of 150 mg/L, TCH concentration of 200 mg/L, and PMS concentration of 300 mg/L. The degradation efficiencies during oxidation processes are shown in Figure 1. The TCH degradation efficiencies were 33%, 50%, 43%, and 41% for catalyst 1, catalyst 2, catalyst 3, and catalyst 4, respectively, at a reaction time of 20 min. It was noticed that the degradation efficiency of catalyst 2 for TCH was higher than that of the other three catalysts. Meanwhile, the catalytic oxidation processes by PMS activated with catalyst 2, MoS_2_, CoFe_2_O_3_, and pretreated steel pickling sludge were also studied and the results are exhibited in Figure S1. As expected, catalyst 2 possessed the best TCH degradation performance compared with solely MoS_2_ and CoFe_2_O_3_.

Two factors might be conducive to enhancing the PMS activation capacity with catalyst 2. One was the incorporation of Co and Fe, as described in our previous study [24]. The other reason was the co-catalytic effect of MoS_2_ and magnetic particles [25]. Hence, the appropriate proportion of cobalt acetate, sodium acetate, and pretreated steel pickling sludge would significantly improve the catalytic activity of the catalyst. Catalyst 2 was selected as the optimal catalyst to describe the structure, influence of operational parameters, and reaction mechanism.

### 2.2. Characterization of the Prepared Catalyst

**Figure 2:**
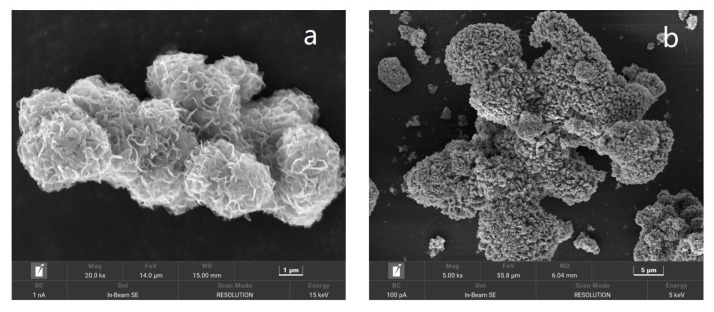
SEM images of the prepared MoS_2_ (**a**) and the prepared catalyst 2 (**b**).

As shown in our previous study, the pristine iron sludge possessed uneven particle size and rough structure [24]. The surface topography and morphology of the prepared MoS_2_ and catalyst 2 were characterized and are presented in Figure 2. As shown in Figure 2a, the prepared MoS_2_ was spherical with a layered sheet-like structure. This special hierarchical structure can increase the spines and porosity distributed on the surface of the microspheres, resulting in a higher surface–volume ratio. After the second solvothermal reaction, many of the nanorods were densely assembled on the surface of the MoS_2_ microspheres, confirming the successful fabrication of the target urchin-like structure, as shown in Figure 2b. The energy dispersive spectrometer (EDS) analysis confirmed that C, N, O, S, Fe, Co, and Mo in the prepared catalyst were uniformly distributed and that Fe, O, and C were the predominant elements in catalyst 2 (as shown in Figure S2), where the wt% of Fe, O, and C was approximately 55:20:15 (as shown in Figure S3 and Table S2). The scanning electron microscopy (SEM) characterization proved that the prepared catalyst 2 had an urchin-like structure.

**Figure 3:**
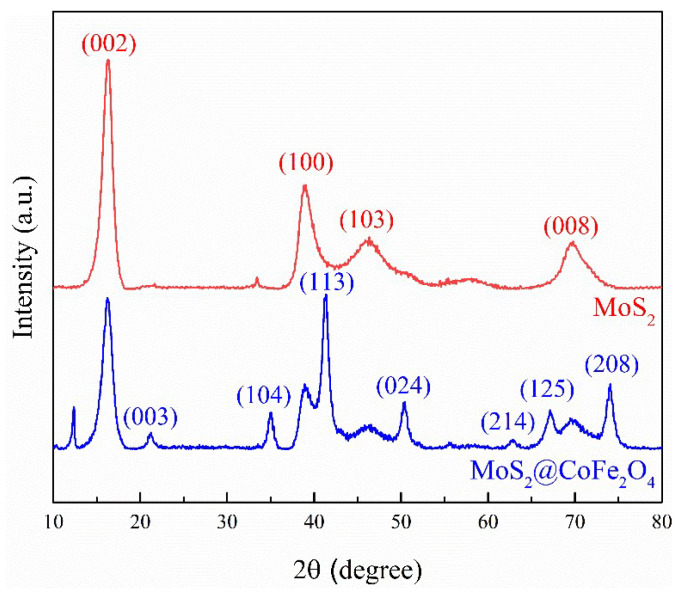
XRD patterns of the prepared MoS_2_ and the prepared catalyst 2.

The X-ray diffraction (XRD) patterns (Figure 3) show that the prepared MoS_2_ displays typical peaks assigned to JCPDS Card no.37-1492, as the characteristic peaks at 16.7°, 38.1°, 46.2°, and 71.1° correspond to (002), (100), (103), and (008) planes, respectively. In the case of catalyst 2 as shown in Figure 3 with the blue line, the characteristic peaks at 21.2°, 35.1°, 41.3°, 50.4°, 62.9°, 67.2°, and 74.1° correspond to (003), (104), (113), (024), (214), (125), and (208) planes, respectively (JCPDS Card no79-1744), indicating that an Fe-based bimetallic catalyst (CoFe_2_O_4_) was loaded on the MoS_2_ layered sheet-like structure. The results of SEM and XRD showed that catalyst 2 was urchin-like structured MoS_2_@CoFe_2_O_4_ derived from steel pickling sludge.

**Figure 4:**
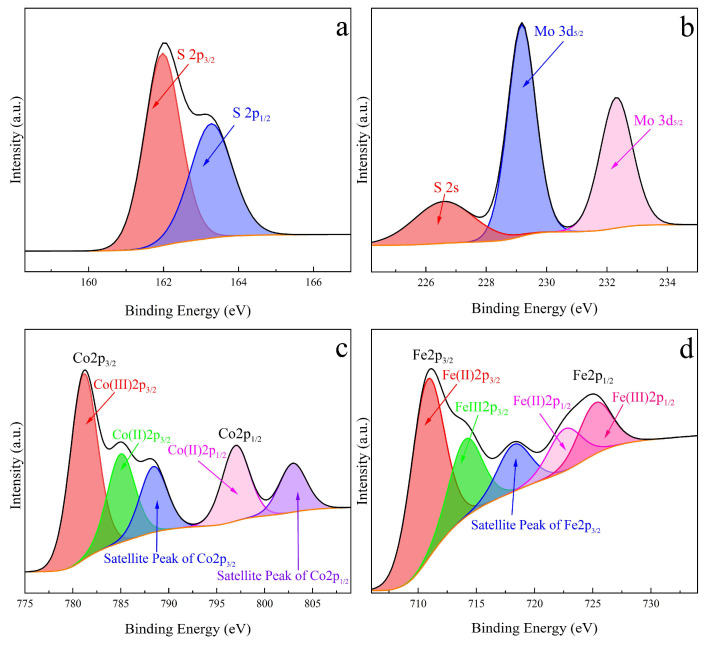
XPS spectra of the prepared MoS_2_@CoFe_2_O_4_: (**a**) S 2p spectrum; (**b**) Mo 3d spectrum; (**c**) Co 2p spectrum; (**d**) Fe 2p spectrum.

X-ray photoelectron spectroscopy (XPS) measurements reveal the chemical characteristics of the prepared MoS_2_ and urchin-like structured MoS_2_@CoFe_2_O_4_. Figure S4a shows the high-resolution S 2p spectra of the MoS_2_, confirming the peaks of 2p_3/2_ (162.3 eV) and 2p_1/2_ (163.5 eV). The spin-orbit discrepancy of 1.2 eV revealed the divalent sulfur in MoS_2_ [26]. Figure S4b depicts Mo 3d spectra, consist of chemically shifted peaks at 229.4 eV and 232.6 eV. The spin–orbit discrepancy of 3.2 eV between 3d_5/2_ (229.4 eV) and 3d_3/2_ (232.6 eV) peaks verified tetravalent molybdenum in MoS_2_ [27]. The peak at lower binding energy (226.8 eV) is on behalf of the S 2s character in MoS_2_. The S 2p and Mo 3d spectra of MoS_2_@CoFe_2_O_4_, as shown in Figure 4a,b, were noted to be identical with the correlation spectra of MoS_2_. Figure 4c verified that Co(II) and Co(III) exist in prepared MoS_2_@CoFe_2_O_4_. The deconvolution of Co 2p spectrum into chemically shifted three peaks at 781.2, 785.1, and 797.1 eV were attributed to Co(III) 2p_3/2_, Co(II) 2p_3/2_, and Co(II) 2p_1/2_, respectively. The existence of the Co(III)/Co(II) redox couple in the catalyst would exhibit excellent electron transport properties for PMS activation. Figure 4d shows the Fe 2p spectra of the MoS_2_@CoFe_2_O_4_, and the deconvolution of the Fe 2p spectrum into four chemically shifted peaks located at 710.8, 714.1, 724.3, and 727.6 eV were Fe(II) 2p_3/2_, Fe(III) 2p_3/2_, Fe(II) 2p_1/2_, and Fe(III) 2p_1/2_, respectively, which was consistent with our previous research [24].

### 2.3. Effects of Operational Parameters on Degradation Efficiency

**Figure 5:**
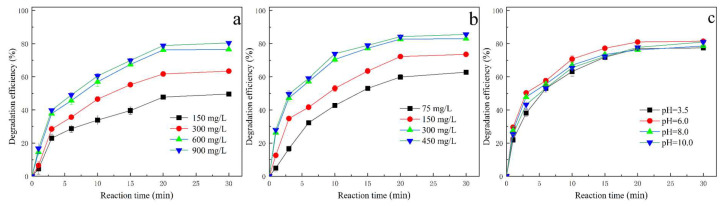
Effects of operational parameters on degradation efficiency: (**a**) the effect of PMS dosage (TCH 200 mg/L, initial solution pH 6.0, and catalyst 150 mg/L); (**b**) the effect of catalyst dosage (TCH 200 mg/L, initial solution pH 6.0, and PMS 600 mg/L); (**c**) the effect of initial pH value (TCH 200 mg/L, PMS 600 mg/L, and catalyst 300 mg/L).

The dosage of PMS is a critical factor in the oxidation process of wastewater treatment, as it directly influences the generation of ROS, which are essential for the degradation of pollutants [28]. Figure 5a demonstrates the impact of varying PMS dosages, from 150 mg/L to 900 mg/L, on the removal efficiency of TCH. At a reaction time of 20 min, the TCH removal efficiencies were observed to be 47.7%, 63.4%, and 76.2% for PMS dosages of 150 mg/L, 300 mg/L, and 600 mg/L, respectively. This demonstrates a significant enhancement in TCH degradation efficiency with increasing PMS dosage. However, when the PMS dosage was further increased to 900 mg/L, the TCH removal efficiency slightly decreased to 78.8%. This decline at higher PMS concentrations can be explained by the recombination of excess active free radicals, as illustrated in Equations (1)–(3) [29]. Moreover, an elevated PMS dosage can enhance the radical quenching effect, which competes with the degradation process, thereby reducing the overall TCH removal efficiency, as detailed in Equations (1)–(7) [30,31].

SO_4_^∙−^ + SO_4_^∙−^ → S_2_O_8_^2−^ (1)

∙OH + ∙OH → H_2_O_2_ (2)

SO_4_^∙−^ + ∙OH → HSO_5_^−^ (3)

SO_4_^∙−^ + HSO_5_^−^ → SO_5_^∙−^ + SO_4_^2−^ + H^+^ (4)

∙OH + HSO_5_^−^ → SO_5_^∙−^ + H_2_O (5)

SO_4_^∙−^ + HSO_5_^−^ + H_2_O → HO_2_^∙−^ + 2SO_4_^2−^ + H^+^ (6)

∙OH + HSO_5_^−^ → HO_2_^∙−^ + 2SO_4_^2−^ + H^+^ (7)

The influence of catalyst dosage on the degradation efficiency of TCH is illustrated in Figure 5b. As the catalyst dosage was incrementally increased from 75 mg/L to 300 mg/L, a notable improvement in TCH degradation efficiency was observed, with TCH removal rates increasing from 59.8% to 82.7% at a reaction time of 20 min, respectively. This trend highlights the positive correlation between catalyst dosage and pollutant degradation efficiency within this range. However, when the catalyst dosage exceeded 450 mg/L, the rate of improvement in TCH removal began to diminish, indicating a saturation effect. Research has consistently shown that in many AOPs, excessive catalyst amounts do not proportionally enhance the reaction rate [32,33]. At lower dosages, the degradation of TCH primarily occurs on the catalyst surface, and increasing the catalyst dosage provides more active sites, thereby improving removal efficiency. However, when the catalyst concentration becomes too high, the availability of reactive species such as PMS and target pollutants like TCH becomes limited, leading to reduced adsorption efficiency and a plateau in the removal rate [34]. Furthermore, other factors such as the dispersion of the catalyst, the accessibility of active sites, and the potential aggregation of catalyst particles at high concentrations can also influence the overall degradation process. For instance, excessive catalyst loading may lead to particle agglomeration, which reduces the effective surface area available for reactions. Additionally, the interaction between the catalyst and oxidants like PMS can be affected by the concentration of both components, as an imbalance may result in incomplete utilization of reactive species. Therefore, optimizing the catalyst dosage is crucial to achieving maximum degradation efficiency while avoiding resource wastage and inefficiencies in the treatment process. This balance ensures that the system operates within the most effective range, maximizing pollutant removal without unnecessary overuse of catalytic materials. Based on a comprehensive analysis of operational parameters, the optimal dosages for the oxidation system were determined as follows: a PMS dosage of 600 mg/L was identified as optimal. Similarly, a catalyst dosage of 300 mg/L was established as optimal. These optimized parameters (600 mg/L PMS and 300 mg/L catalyst) collectively ensure efficient degradation, resource efficiency, and operational practicality for TCH removal.

The initial pH of the solution plays a significant role in influencing the oxidation process, making it essential to evaluate its impact to optimize operational conditions. Figure 5c demonstrates the effect of varying initial pH levels, ranging from 3.5 to 10.0, on the removal efficiency of TCH. The results indicate that the degradation efficiency of TCH is relatively unaffected by changes in pH, suggesting a broad operational pH range for the process. This behavior can be attributed to the properties of PMS, which exists primarily as HSO_5_^−^ under acidic and neutral conditions, given its pK_a_ values (pK_a1_ < 0; pK_a2_ = 9.4) [35]. In these conditions, the formation of hydrogen bonds between the O-O group in HSO_5_^−^ and H^+^ ions can stabilize the PMS molecule. However, this stabilization may hinder its electrostatic interaction with positively charged bimetallic catalysts, potentially affecting the overall reaction dynamics [36]. Additionally, an excessive concentration of H^+^ in highly acidic environments can scavenge oxidative free radicals, as illustrated in Equations (8) and (9), thereby reducing the availability of reactive species for pollutant degradation [37,38]. Beyond these factors, the pH of the solution can also influence the surface charge of the catalyst and the ionization state of the target pollutant, both of which play critical roles in the degradation process. For instance, at higher pH levels, the catalyst surface may become negatively charged, potentially repelling negatively charged intermediates or byproducts, while at lower pH levels, the protonation of functional groups on the catalyst or pollutant may alter their reactivity.

SO_4_^∙−^ + H^+^ + e^−^→ HSO_4_^∙−^ (8)

∙OH + H^+^ + e^−^ → H_2_O (9)

Although TCH was completely eliminated by the MoS_2_@CoFe_2_O_4_ catalyst/PMS system, the efficacy of AOPs for organic pollutant removal is conventionally measured by the decrease in Chemical Oxygen Demand (COD). Therefore, we assessed the COD oxidation ability of the MoS_2_@CoFe_2_O_4_ catalyst under conditions of 200 mg/L TCH, 600 mg/L PMS, and an initial pH of 6.0. As shown in Figure S5, approximately 45% COD removal was attained after 30 min of oxidation. This level of COD suggests that TCH was degraded and oxidized more efficiently during the oxidation process.

### 2.4. Identification of Reactive Species and Plausible Mechanism

Numerous studies have demonstrated that PMS-based AOPs involved multiple degradation pathways, including both radical (SO_4_^∙−^, ∙OH, and O_2_^∙−^) and non-radical (^1^O_2_ and mediated electron transfer) mechanisms [39,40]. To elucidate the dominant reaction pathways in the MoS_2_@CoFe_2_O_4_ catalyst/PMS system, specific quenching experiments were conducted using various ROS scavengers.

Four commonly used radical quenchers were employed at concentrations 50-fold higher than the target pollutant to ensure complete ROS scavenging: methanol (MeOH) for SO_4_^∙−^ (rate constant *k* = 2.5 × 10^7^ M^−1^ s^−1^) and ∙OH (*k* = 9.7 × 10^8^ M^−1^ s^−1^), tert-butanol (TBA) for ∙OH (*k* = 3.8–7.6 × 10^8^ M^−1^ s^−1^), chloroform (CF) for O_2_^∙−^ (*k* = 3 × 10^10^ M^−1^ s^−1^), and furfuryl alcohol (FFA) for singlet oxygen (^1^O_2_) [41]. These quenching experiments helped distinguish the contributions of different ROS in the degradation process. By systematically analyzing the inhibition effects of these scavengers, this study aimed to identify the primary ROS responsible for pollutant degradation, providing deeper insights into the underlying reaction mechanisms.

**Figure 6:**
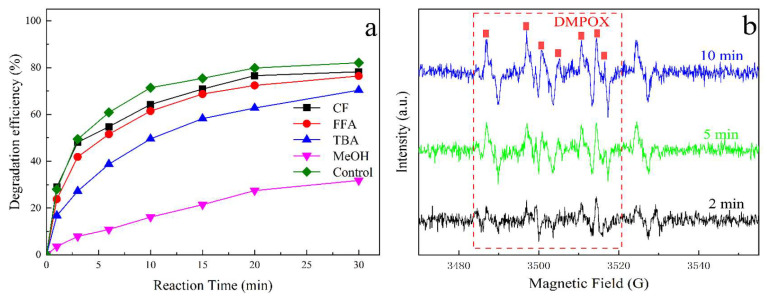
(**a**) Effects of radical scavengers on degradation efficiency (TCH 200 mg/L, PMS 600 mg/L, catalyst 300 mg/L, and initial solution pH 6.0); (**b**) DMPO-trapping EPR signals with different reaction times.

Figure 6a illustrates the influence of different quenchers on the degradation efficiency of TCH in the oxidation system. Since the prepared MoS_2_@CoFe_2_O_4_ catalyst is an iron-based bimetallic composite, it primarily promotes the generation of SO_4_^∙−^ and ∙OH during the PMS activation process, as reported in previous studies [42]. To distinguish the contributions of these radicals, MeOH and TBA were employed as radical scavengers. The experimental results revealed a significant difference in their quenching effects: MeOH exhibited a stronger inhibitory effect on degradation efficiency compared to TBA, with oxidation efficiencies of 31% and 70% for TCH, respectively, after 30 min of reaction. This observation suggests that SO_4_^∙−^ played a dominant role in the degradation process, while ∙OH contributed to a lesser extent.

To further verify the presence of these reactive species, electron paramagnetic resonance (EPR) spectroscopy coupled with 5,5-dimethyl-1-pyrroline N-oxide (DMPO) spin trapping was conducted. The EPR spectra (Figure 6b) revealed a characteristic seven-line signal pattern. This spectral signature can be attributed to the formation of 5-tert-butoxycarbonyl-5-methyl-2-oxo-pyrroline-1-oxyl (DMPOX), a stable nitroxide radical that exhibits analogous characteristics to the well-documented DMPOX species reported in previous studies [43]. Notably, the absence of detectable BMPO-OH or BMPO-SO_4_ adducts in the spectra suggests alternative reaction pathways. For the MoS_2_@CoFe_2_O_4_ catalyst, temporal analysis showed dynamic changes in BMPOX signal intensity, with the intensity of the characteristic peaks gradually increased from 2 min to 10 min. The formation of BMPOX may originate from the nucleophilic attack of SO_4_^∙−^ on the BMPO molecule, followed by subsequent oxidation and rearrangement processes. This interpretation is supported by previous reports demonstrating the challenges in detecting transient DMPO-SO_4_ adducts due to their inherent instability and limited detection sensitivity [44]. The observed BMPOX signal could potentially arise through two possible mechanisms: (1) direct reaction between BMPO and SO_4_^∙−^, or (2) secondary transformation of initially formed BMPO-SO_4_ adducts. The latter pathway appears more plausible considering the well-documented instability of sulfate radical adducts in spin trapping experiments. These findings provide valuable insights into the complex radical transformation processes occurring in the oxidation system, highlighting the importance of Co modification in promoting radical generation and stabilization.

**Figure 7:**
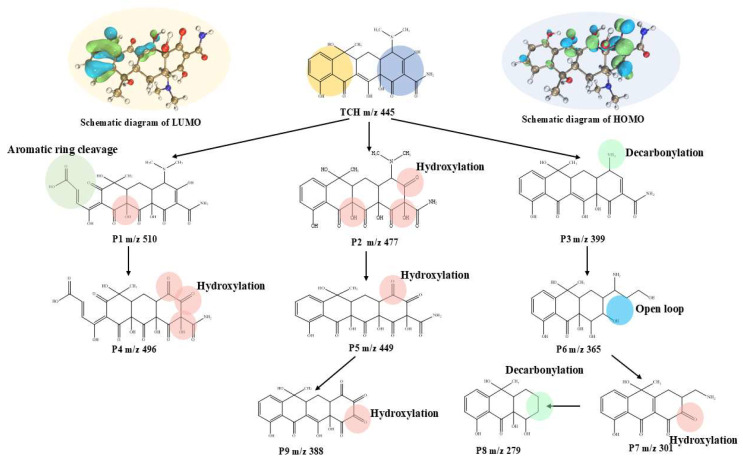
Proposed pathways for the oxidative degradation of TCH in the MoS_2_@CoFe_2_O_4_ catalyst/PMS system, with the TCH chemical structure, LUMO and HOMO diagrams.

DFT calculations employing the B3LYP/6-31G (d,p) method were conducted to investigate the charge distribution of TCH via natural population analysis (NPA), aiming to elucidate the degradation mechanism in the MoS_2_@CoFe_2_O_4_ catalyst/PMS system (detailed procedures: Text S3 and Figure S6). Figure 7 presents the TCH chemical structure and LUMO and HOMO diagrams. The detailed NPA charges and Fukui indices (f^+^, f^−^) are shown in Table S3. The LUMO highlights electron-accepting regions (blue), while the HOMO highlights electron-donating regions (green). To elucidate the degradation pathways and reaction mechanism of TCH in the MoS_2_@CoFe_2_O_4_ catalyst/PMS system, HPLC-MS/MS was employed to identify intermediates (detailed procedures: Text S4 and Table S4). TCH possesses electron-rich functional groups—including double bonds, aromatic rings, phenolic hydroxyl, and amino groups—making it susceptible to attack by ROS during degradation. Based on the identified reaction products, this study proposes a potential reaction mechanism for TCH degradation in this process. This mechanism primarily involves three distinct pathways, hydroxylation, decarbonylation, and ring-opening, as illustrated in Figure 7. The hydroxylation reaction involved substitution of a hydrogen atom on tetrahydrobenzene within the TCH molecule by a hydroxyl group. Analysis detected product P1 was produced via epoxidation of the double bonds, inducing hydroxylation and aromatic ring cleavage. P2 was produced via ∙OH attack on TCH’s C=C, inducing hydroxylation. Dealkylation and functional group removal reactions were identified: P3 resulted from N-demethylation. Products P4, P5, and P9 likely formed through SO_4_^∙−^-mediated hydroxylation. P6, P7, and P8 potentially arose from carbonyl and aminomethyl elimination and ring-opening reactions featuring intramolecular cleavage, fragmenting TCH into smaller molecules. This reaction pathway was frequently observed in TCH degradation [45,46].

**Figure 8:**
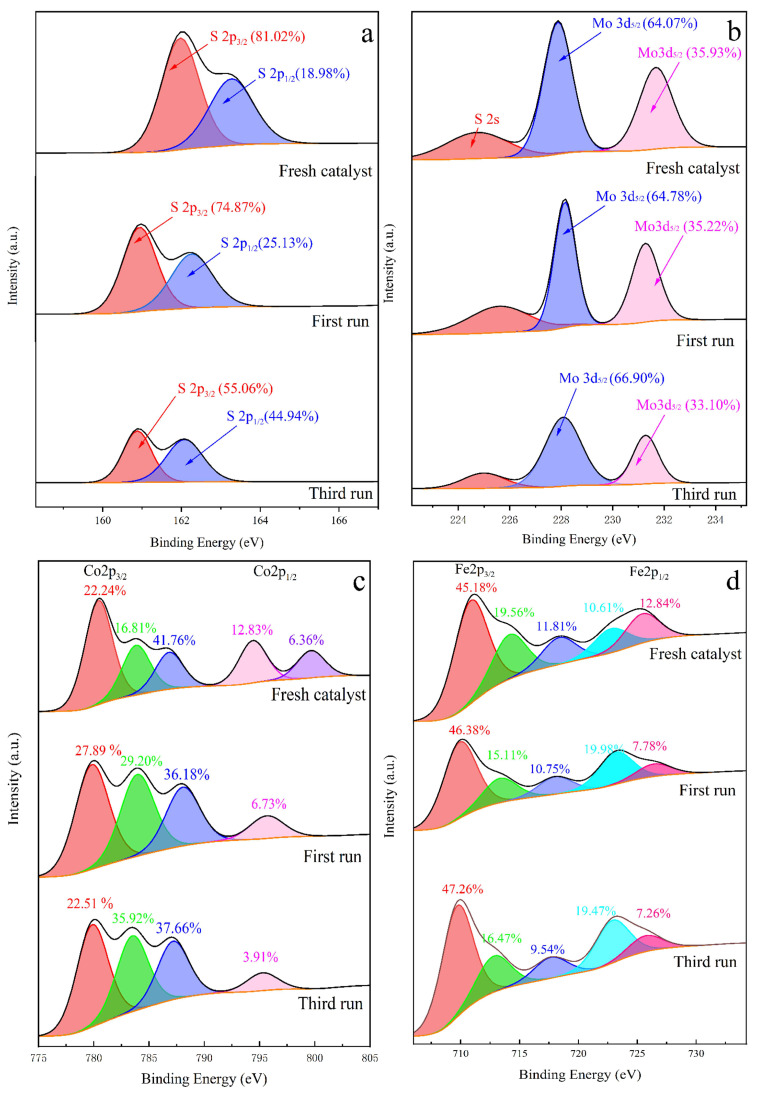
XPS spectra of the prepared MoS_2_@CoFe_2_O_4_: (**a**) S 2p spectrum; (**b**) Mo 3d spectrum; (**c**) Co 2p spectrum; (**d**) Fe 2p spectrum after first and third run.

As evidenced by XPS survey spectra, S, Mo, Co, and Fe were detected on fresh and used catalyst surfaces. Their respective high-resolution spectra are presented in Figure 8. The S 2p spectra, as depicted in Figure 8a, suggested that the doping effect causes local changes in electron density during the reaction process, resulting in slight shifts of certain S 2p peaks and affecting the overall proportion [47]. The variation in changed electron density provides electrons for the valence state alteration of iron and cobalt elements in the catalyst. As shown in Figure 8b, the Mo 3d spectra indicated that the main state of molybdenum element was Mo(VI), where the ratios of each peak remained basically unchanged during the reaction process [48]. To elucidate the synergistic effects and distinct functions of Fe and Co species in the prepared catalyst during PMS activation, the respective high-resolution spectra of Fe 2p and Co 2p were analyzed, with the relevant results presented in Figure 8c,d. It is noteworthy that the Co 2p3/2 spectra revealed distinct changes in the cobalt valence states on fresh and used catalysts. The proportion of Co(II) in the post-reaction catalyst shows a significant increase, indicating the reduction of some Co(III) to Co(II) during the catalytic process. This phenomenon originates from the spontaneous electron transfer between MoS_2_ and Co(III) [49]. The high-resolution Fe 2p spectra revealed four characteristic peaks corresponding to different oxidation states: the doublet at 710.8 eV (Fe(II) 2p3/2) and 724.3 eV (Fe(II) 2p1/2), along with the pair at 714.1 eV (Fe(III) 2p3/2) and 727.6 eV (Fe(III) 2p1/2). Quantitative analysis demonstrated a remarkable increase in the Fe(II)/Fe(III) ratio in the used catalyst compared to the fresh sample, strongly suggesting that Fe(III) sites undergo reductive transformation to Fe(II) during the PMS activation process. The distinct behavior of these transition metals in the supported catalyst provides valuable insights into the reaction mechanism, where Fe(II)/Fe(III) and Co(II)/Co(III) redox cycling appears crucial for PMS activation, while MoS2 provides sufficient electrons for these redox cycling within the catalyst framework, as illustrated in Equations (10)–(12) [50].

Co^2+^/Fe^2+^ + HSO_5_^−^ → Co^3+^/Fe^3+^ + SO_4_^∙−^ + OH^−^ (10)

Co^2+^/Fe^2+^ + HSO_5_^−^ → Co^3+^/Fe^3+^ + ∙OH + SO_4_^2−^ (11)

e^−^ + Co^3+^/Fe^2+^ → Co^2+^/Fe^3+^ (12)

### 2.5. Stability of the MoS_2_@CoFe_2_O_4_ Catalyst

**Figure 9:**
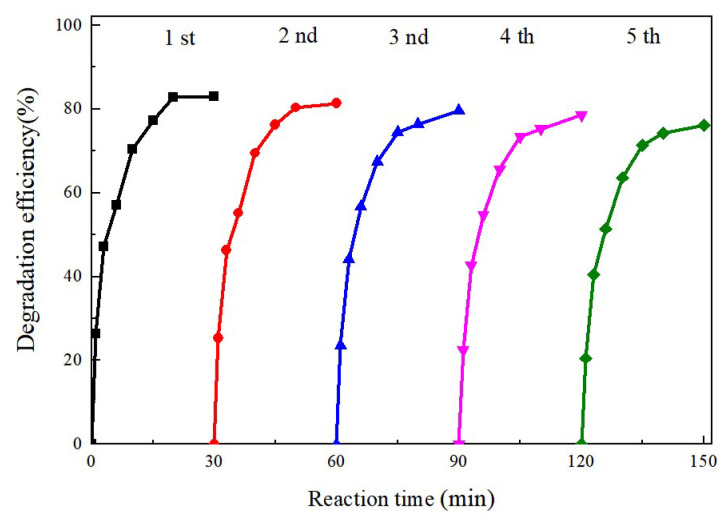
Reusability experiments of MoS_2_@CoFe_2_O_4_ catalyst for the degradation of TCH (TCH 200 mg/L, PMS 600 mg/L, catalyst 300 mg/L, and initial solution pH 6.0).

Catalyst stability and reusability are critical factors in the process of practical application. To evaluate these properties of the synthesized MoS_2_@CoFe_2_O_4_ catalyst, five consecutive recycling tests were conducted under the same conditions. After each run, the catalyst was recovered via extraction and filtration. As illustrated in Figure 9, the TCH degradation efficiency remained high (>75%) across all cycles, with slight declines from 82% (first run) to 76% (fifth run) after 30 min of reaction. This minor reduction may be attributed to inevitable catalyst loss during recovery.

The reusability tests confirmed the robust stability and sustained catalytic activity of the MoS_2_@CoFe_2_O_4_ catalyst. Metal leaching was monitored by ICP-OES/MS, revealing negligible release of iron and cobalt ions (Table S5), which underscores the catalyst’s structural integrity. Post-reaction characterization (SEM) further verified its stability: the layered morphology was preserved (Figure S7). These results highlight the effectiveness of the solvothermal synthesis in producing a durable AOP catalyst.

To investigate the operational stability of catalysts in actual water bodies, we collected effluent from the secondary sedimentation tank of Linyi University Town Wastewater Treatment Plant to prepare corresponding solutions. Analyses of the effluent characteristics are presented in Table S6. Under continuous-flow conditions in these aqueous environments, the release of iron and cobalt ions is shown in Table S7. The catalysts maintained relatively high catalytic activity and stability in actual water bodies.

## 3. Materials and Methods

### 3.1. Materials

The chemicals used in the solvothermal synthesis, including thiourea (CH_4_N_2_S, 99%), ammonium molybdate tetrahydrate ((NH_4_)_6_Mo_7_O_24_·4H_2_O, 99%), cobalt acetate (GR), sodium acetate (AR), and ethylene glycol (GR), were procured from Sinopharm Chemical Reagent Beijing Co., Ltd. (Beijing, China). Tetracycline hydrochloride (TCH, C_22_H_24_N_2_O_8_·HCl, >98%) was purchased from TCI (Shanghai, China) and used without any further purification. Peroxymonosulfate (PMS, Oxone, KHSO_5_·0.5 KHSO_4_·0.5 K_2_SO_4_, AR), used as an oxidizing agent, was obtained from Sinopharm Chemical Reagent Beijing Co., Ltd. (Beijing, China). Methanol (MeOH), tert-butanol (TBA), furfuryl alcohol (FFA), and chloroform (CF) were obtained as HPLC-grade from Sinopharm Chemical Reagents Co., Ltd. (Shanghai, China). These compounds were used as radical scavengers in the study. All other reagents used in this research were purchased from Sinopharm Chemical Reagents Co., Ltd. (Shanghai, China). Ultrapure water with a resistivity of 18.2 MΩ·cm obtained from the Milli-Q system (Merck KGaA, Darmstadt, Germany) was utilized for preparing all solutions in this investigation. The iron sludge obtained from the Wanfang metal sheet metal processing plant (Shandong, China) was pretreated and analyzed; the treatment process details are listed in Text S1.

### 3.2. Preparation of the Catalysts

A convenient two-step solvothermal method was utilized to develop the magnetic urchin-like structured MoS_2_@CoFe_2_O_4_ by recycling steel pickling sludge as the Fe source. Firstly, 1000 mg thiourea and 500 mg (NH_4_)_6_Mo_7_O_24_·4H_2_O were added into 50 mL ultrapure water. The mixed solution, stirred thoroughly for 10 min, was transferred into a 100 mL sealed Teflon-lined stainless-steel reaction kettle for solvothermal synthesis under 220 °C for 720 min. After chilling overnight, MoS_2_, the ultramarine precipitate, was collected by suction filtration and washed several times with ultrapure water. Then, the prepared MoS_2_ was dried in a vacuum oven at 60 °C for 300 min. In the second step, 100 mg prepared MoS_2_ powder and fixed amounts of cobalt acetate, sodium acetate, and pretreated steel pickling sludge were added into 40 mL ethylene glycol (as listed in Table S1). For a clear description, the catalysts synthesized with different proportions were marked as catalyst 1, catalyst 2, catalyst 3, and catalyst 4, respectively. CoFe_2_O_3_ was prepared by the addition of 120 mg cobalt acetate, 200 mg sodium acetate, and 200 mg pretreated steel pickling sludge. After 30 min mechanical stirring, the mixed liquor was moved into a 100 mL sealed Teflon-lined stainless-steel reaction kettle for solvothermal synthesis under 200 °C for 600 min with heating rate setting at 5 °C/min. After chilling overnight, MoS_2_@CoFe_2_O_4_ was collected by suction filtration and washed several times with methanol and ultrapure water. Then, the prepared catalysts were dried in a vacuum oven at 60 °C for 300 min. This method of obtaining the catalyst operates at moderate temperatures without high-temperature calcination or complex post-treatments. The ethylene glycol solvent enables lower temperatures than solid-state synthesis, while the utilization of waste sludge eliminates costly metal purification. This approach transforms industrial sludge into high-value catalysts with minimal synthesis complexity.

### 3.3. Degradation of Tetracycline Hydrochloride

Degradation experiments were performed in a homemade plexiglass reactor with 100 mL volume. During the oxidation process, the reaction mixture was mixed thoroughly with a Teflon rod at a thermostatically controlled temperature (25 ± 2 °C). Before the reaction started, the catalyst was added into TCH solution, followed by the PMS to trigger the oxidation reaction. The pH value of reaction mixture was adjusted by 1 M NaOH or H_2_SO_4_ solution. Samples taken from reactor at set intervals were filtered through a 0.2 μm polyether sulfone filter for subsequent analysis.

### 3.4. Analytic Methods and Characterization

The prepared urchin-like structures of MoS_2_@CoFe_2_O_4_ were characterized by scanning electron microscopy (SEM), X-ray photoelectron spectroscopy (XPS), X-ray diffraction (XRD), and a specific surface and porosity analyzer. The details of these tests are presented in Text S2.

The concentration of tetracycline hydrochloride in the filtered solution was detected by a Hach DR-6000 Spectrophotometer (Hach, Loveland, CO, USA) by the established standard with wavelength set at 357 nm.

## 4. Conclusions

This study successfully synthesized supported MoS_2_@CoFe_2_O_4_ catalyst from steel pickling sludge via a facile solvothermal method, demonstrating an efficient and sustainable approach for waste-to-resource conversion. The as-prepared catalyst exhibited exceptional PMS activation capability, achieving rapid degradation of TCH under TCH 200 mg/L, PMS 600 mg/L, and catalyst dosage 300 mg/L. Notably, the catalyst maintained high degradation efficiency across a wide range of initial pH values. Mechanistic investigations, including quenching experiments and EPR analysis, confirmed that SO_4_^∙−^ played a dominant role in the oxidative degradation process. The catalyst demonstrated remarkable stability and reusability over multiple cycles, with minimal metal leaching (Fe, Co, and Mo), as confirmed by ICP-MS analysis. A synergistic effect between MoS_2_ and CoFe_2_O_4_ was identified, enhancing PMS activation and improving catalytic performance. Intermediates of TCH degradation were identified during the MoS_2_@CoFe_2_O_4_ catalyst/PMS process, revealing four potential pathways: hydroxylation, decarbonylation, ring-opening, and oxidation reactions. This study not only presents a cost-effective strategy for valorizing industrial waste (pickling sludge) but also provides a highly efficient, environmentally friendly bimetallic catalyst for AOPs in wastewater treatment.

## Data Availability

Data are contained within the article.

## Supplementary Materials

The following supporting information can be downloaded at https://www.mdpi.com/article/10.3390/molecules30153194/s1, Text S1: Pretreatment process of steel pickling sludge; Text S2: Characterization of the catalyst; Text S3: DFT approach; Text S4: Product detection methods; Table S1: Dosage of each component in the catalysts synthesis processes; Table S2: Elemental composition of the catalyst 2; Table S3: The detailed NPA charge distribution and Fukui index of TCH; Table S4: The m/z values, mass spectra, and proposed structural formulas of TCH and its intermediate products; Table S5: The concentrations of Fe, Co, and Mo in the working solutions after each trial; Table S6: Characterization of the effluent; Table S7: The concentrations of Fe, Co, and Mo in the effluent solutions after each trial; Figure S1: Effect of different catalysts on degradation efficiency (initial pH value of 3.7, catalyst concentration of 200 mg/L, tetracycline hydrochloride of 200 mg/L, PMS concentration of 400 mg/L, reaction time of 20 min); Figure S2: EDX mapping image of catalyst 2; Figure S3: EDS spectra of catalyst 2; Figure S4: XPS spectra of the prepared MoS_2_: (a) S 2p spectrum in MoS_2_; (b) Mo 3d spectrum in MoS_2_; Figure S5: COD removal of TCH solution during oxidation process; Figure S6: Molecular structural formula of TCH; Figure S7: SEM images of the CoFe_2_O_4_ layered catalyst after fifth cycle.
