# Supplementary material for: Efficient Tetracycline Hydrochloride Degradation by Urchin-Like Structured MoS2@CoFe2O4 Derived from Steel Pickling Sludge via Peroxymonosulfate Activation
Source: Molecules. 2025 Jul 30;30(15):3194. doi: 10.3390/molecules30153194 (PMC12348658; doi:10.3390/molecules30153194)

Efficient tetracycline hydrochloride degradation by urchin-like structured $MoS_2@CoFe_2O_4$ derived from steel pickling sludge via peroxymonosulfate activation

Jin Qi[1], Kai Zhu[1],*, Ming Li[1], Yucan Liu[2], Pingzhou Duan[3] and Lihua Huang[1],*

[1] *Shandong Provincial Key Laboratory of Water and Soil Conservation and Environmental Protection, College of resources and environment, Linyi University, Linyi, 276000, China*

[2] *School of Civil Engineering, Yantai University, Yantai, 264005, China*

[3] *State Key Laboratory of Environmental Criteria and Risk Assessment, Chinese Research Academy of Environmental Sciences, Beijing 100012, China*

Corresponding author:

Kai Zhu

E–mail: zhukai@lyu.edu.cn

Lihua Huang

E–mail: huanglihua@lyu.edu.cn

Text S1. Pretreatment process of steel pickling sludge

The iron sludge used in this research was acquired from Wanfang metal sheet metal processing plant (Shandong, China), with HCl was used as the pickling solution. The iron sludge was obtained during the neutralization process of pickling waste liquid by $CaCO_3$. The collected dehydrated iron sludge was first washed with tap water several times to remove impurities, and then dried in a 105°C constant temperature oven Subsequently, the dried steel pickling sludge is crushed, ground, screened through a 200-mesh sieve and stored in a dryer at room temperature for further use.

Text S2. Characterization of the catalyst

The obtained SEM images were recorded by a Tescan Mira Lms microscope equipped with an Energy Dispersive Spectrometer (EDS). The XPS characterization was recorded by an ESCALAB 250XI spectrometer (ThermoFisher, USA) fitted with a monochromated Al Kalph 150W X-ray source. XRD (D8 Advance, Bruker) with Co radiation was used for the detection of the crystal structure of prepared catalysts. The specific surface area and pore size distribution were analyzed by nitrogen adsorption/desorption isotherms at 393 K with a specific surface area and pore size analyzer (ASAP 2460, MiCROMERITICS, USA).

Text S3. DFT approach

All theoretical calculations in this study were performed using Gaussian 16. Geometry optimizations and frequency calculations were conducted at the B3LYP/6-31G** level of theory. To ensure accuracy, single-point energy calculations were subsequently performed on the optimized structures using the M06-2X functional combined with the def2TZVP basis set. For transition states (TS), frequency analysis confirmed the presence of exactly one imaginary frequency. Given that the reaction occurs in aqueous solution, the SMD implicit solvation model was employed in all energy calculations. Finally, the Fukui function was computed using Multiwfn 3.8(dev) and visualized with VMD.

Text S4. Product detection methods

ACQUITY™ UPLC BEH C18 Column (2.1 mm 100 mm 1.7 μm). Mobile phase: Acetonitrile (A) and 0.1% formic acid in water (B). Flow rate: 0.2 mL/min. Injection volume: 10 μL.

LC-MS Full Scan Mode Parameters and Conditions:

Simultaneous ESI+ and ESI- scanning in full scan mode. m/z range: 50 to 500. Scan time: 100 ms. Capillary voltage: 3 kV. Cone voltage: 25 V. Desolvation temperature: 350 ˚C. Source temperature: 120 ˚C. Desolvation gas flow (99.999% N_2): 500 L/h. Cone gas flow (99.999% N_2): 50 L/h.

Mobile Phase Gradient Program:

0.0 to 1.0 min: 5% A (95% B);

1.0 to 8.0 min: linear gradient from 5% to 75% A (95% to 25% B);

8.0 to 22.0 min: linear gradient from 75% to 100% A (25% to 0% B);

22.0 to 23.0 min: 100% A (0% B);

23.0 to 23.1 min: linear gradient from 100% to 5% A (0% to 95% B);

23.1 to 26.0 min: 5% A (95% B).

Table S1. Dosage of each component in the catalysts synthesis processes

Component	Catalyst 1	Catalyst 2	Catalyst 3	Catalyst 4
MoS_2 (mg)	100	100	100	100
Cobalt acetate (mg)	60	120	180	240
Sodium acetate (mg)	100	200	300	400
Steel pickling sludge(mg)	100	200	300	400

Table S2. Elemental Composition of the catalyst 2

Element	C	N	O	S	Fe	Co	Mo
Wt (%)	15.81	1.44	19.42	1.93	55.53	4.68	1.19
At (%)	34.83	2.72	32.12	1.59	26.31	2.10	0.33

Table S3. The details NPA charge distribution and Fukui index of TCH

Atom	q(N)	q(N+1)	q(N-1)	f-	f+	f0
1(C)	0.1299	0.0742	0.1321	0.0022	0.0556	0.0289
2(C)	-0.0646	-0.0829	-0.0569	0.0077	0.0184	0.013
3(C)	0.138	0.0786	0.1443	0.0062	0.0594	0.0328
4(C)	0.0244	0.02	0.0253	0.0009	0.0045	0.0027
5(C)	-0.0219	-0.025	-0.0202	0.0017	0.0031	0.0024
6(C)	-0.0487	-0.0535	-0.0439	0.0047	0.0048	0.0048
7(C)	-0.0168	-0.0192	-0.0126	0.0042	0.0024	0.0033
8(C)	0.0872	0.0859	0.089	0.0017	0.0013	0.0015
9(C)	-0.004	-0.0212	0.0151	0.0191	0.0173	0.0182
10(C)	-0.065	-0.085	0.0048	0.0697	0.02	0.0449
11(C)	-0.0279	-0.0718	0.0082	0.0361	0.0439	0.04
12(C)	-0.0545	-0.0825	-0.0069	0.0476	0.0281	0.0378
13(C)	0.0967	0.0758	0.1413	0.0445	0.0209	0.0327
14(C)	-0.0426	-0.0483	-0.003	0.0396	0.0057	0.0226
15(C)	0.13	0.0763	0.1378	0.0077	0.0537	0.0307
16(C)	-0.0481	-0.0668	-0.0098	0.0383	0.0187	0.0285
17(C)	0.0936	0.0621	0.1286	0.0349	0.0315	0.0332
18(C)	0.0684	0.0593	0.0708	0.0024	0.0091	0.0058
19(C)	0.1513	0.1445	0.1718	0.0205	0.0068	0.0136
20(O)	-0.3168	-0.3456	-0.2353	0.0815	0.0288	0.0551
21(N)	-0.1498	-0.1634	-0.1241	0.0257	0.0137	0.0197
22(O)	-0.2262	-0.2848	-0.2048	0.0213	0.0587	0.04
23(O)	-0.2324	-0.2931	-0.2191	0.0133	0.0607	0.037
24(C)	-0.0911	-0.0972	-0.0844	0.0067	0.0061	0.0064
25(O)	-0.2135	-0.2401	-0.1982	0.0153	0.0266	0.0209
26(O)	-0.1536	-0.1832	-0.1131	0.0406	0.0296	0.0351
27(O)	-0.1904	-0.2195	-0.1021	0.0883	0.0291	0.0587
28(O)	-0.2062	-0.2101	-0.196	0.0102	0.0039	0.0071
29(O)	-0.1283	-0.1789	-0.1127	0.0156	0.0506	0.0331
30(N)	-0.0482	-0.0497	-0.0421	0.0061	0.0015	0.0038
31(C)	-0.0353	-0.0407	-0.0313	0.004	0.0054	0.0047
32(C)	-0.0328	-0.0384	-0.0279	0.0049	0.0056	0.0053
33(H)	0.0458	0.0366	0.0453	-0.0005	0.0093	0.0044
34(H)	0.0329	0.0216	0.0431	0.0103	0.0113	0.0108
35(H)	0.0242	0.0169	0.0303	0.0061	0.0074	0.0067
36(H)	0.0342	0.022	0.0446	0.0104	0.0122	0.0113
37(H)	0.0326	0.0226	0.0452	0.0126	0.01	0.0113
38(H)	0.0335	0.0182	0.062	0.0285	0.0153	0.0219
39(H)	0.0449	0.0196	0.0717	0.0269	0.0253	0.0261
40(H)	0.0474	0.0268	0.0753	0.0279	0.0206	0.0243
41(H)	0.0926	0.0856	0.1046	0.012	0.007	0.0095

42(H)	0.1253	0.1059	0.1434	0.0181	0.0195	0.0188
43(H)	0.0305	0.0199	0.0397	0.0092	0.0107	0.0099
44(H)	0.0316	0.0238	0.0442	0.0125	0.0078	0.0102
45(H)	0.0359	0.0279	0.0438	0.0079	0.008	0.008
46(H)	0.1373	0.123	0.1464	0.009	0.0143	0.0117
47(H)	0.1391	0.1256	0.155	0.0158	0.0136	0.0147
48(H)	0.1161	0.1055	0.1361	0.02	0.0106	0.0153
49(H)	0.1494	0.1393	0.1578	0.0083	0.0101	0.0092
50(H)	0.1249	0.1096	0.1325	0.0076	0.0153	0.0115
51(H)	0.0422	0.0386	0.0445	0.0024	0.0035	0.003
52(H)	0.0375	0.0238	0.0489	0.0113	0.0138	0.0126
53(H)	0.0248	0.0207	0.0258	0.001	0.0041	0.0025
54(H)	0.042	0.0381	0.0463	0.0043	0.0039	0.0041
55(H)	0.0424	0.0284	0.0533	0.0109	0.014	0.0124
56(H)	0.0317	0.0245	0.036	0.0042	0.0072	0.0057

Table S4. The m/z values, mass spectra and proposed structural formulas of TCH and its intermediate products

Number	m/z	MS/MS	Proposed structural formulas
TCH	445 （M+）	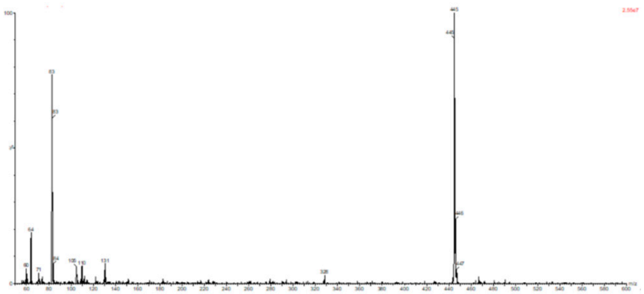	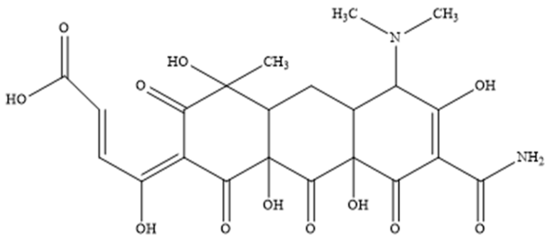
P1	510 （M+）	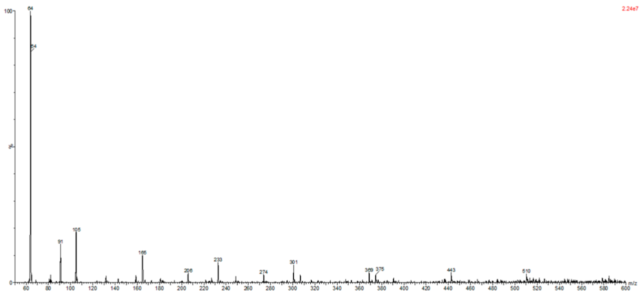	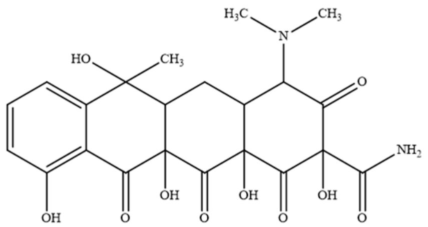

Number	m/z	MS/MS	Proposed structural formulas
P2	477<br>（M+）	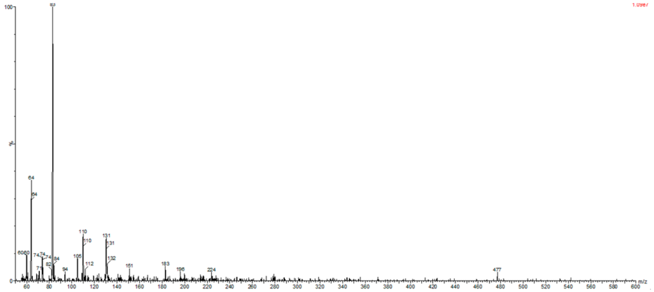	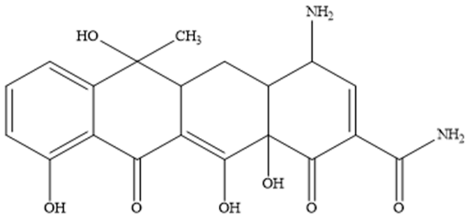
P3	399<br>（M+）	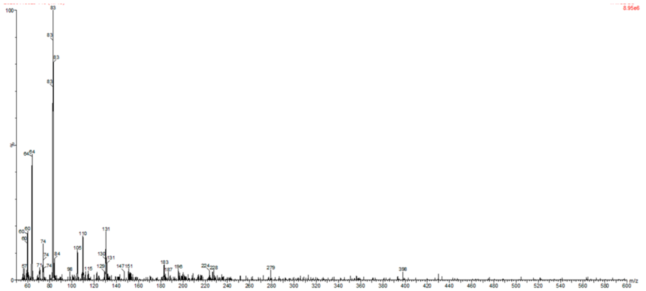	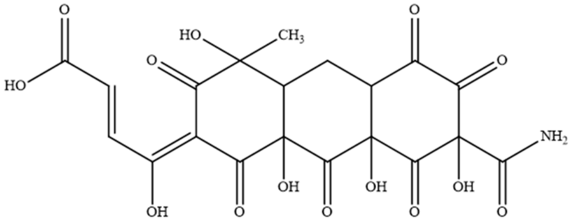
P4	496<br>（M+）	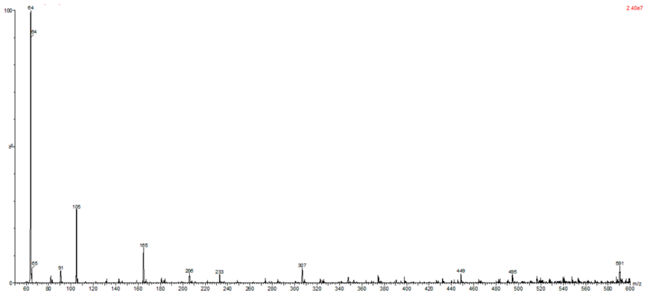	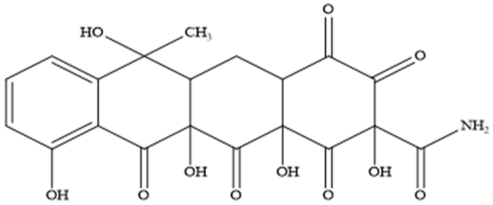

Number	m/z	MS/MS	Proposed structural formulas
P6	351.3 （M+）	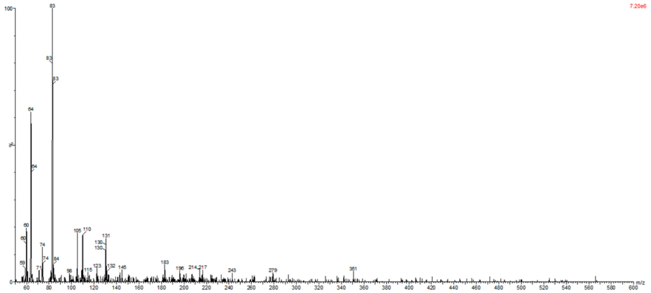	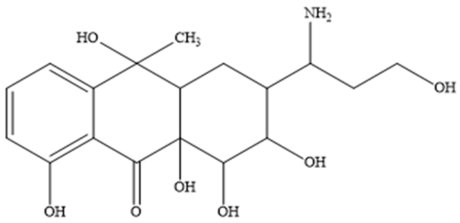
P7	331.1 （M+）	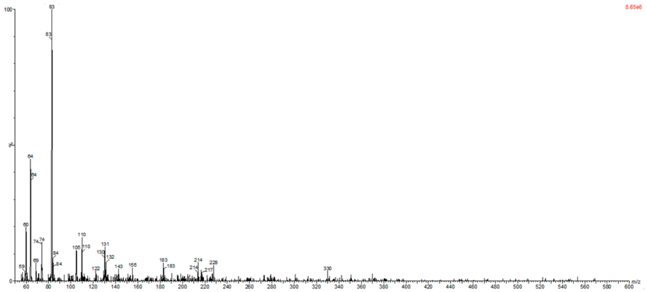	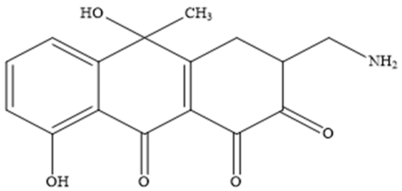
P8	224.5 （M+）	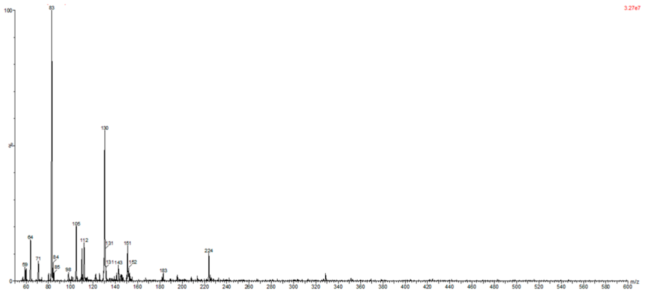	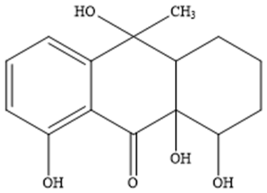

Number	m/z	MS/MS	Proposed structural formulas
P9	225.9 （M+）	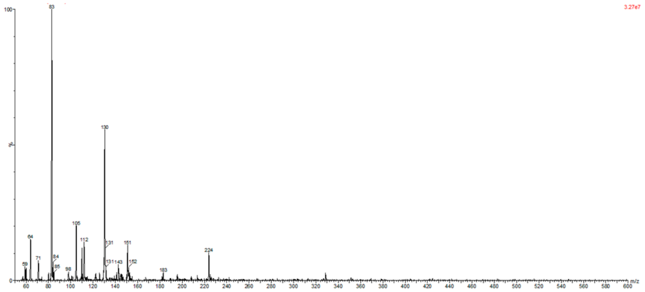	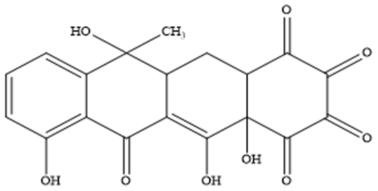

Table S5. The concentrations of Fe, Co and Mo in the working solutions after each trial

Cycle number	Fe (μg/L)	Co (μg/L)	Mo (μg/L)
1	236	281	148
2	327	312	275
3	383	372	264
4	419	487	318
5	348	412	287

Table S6. Characterization of the effluent

Parameters	Effluent
Color	None
Odor	None
pH	6.8
COD	29
NH_3-N	1.7

Table S7. The concentrations of Fe, Co and Mo in the effluent solutions after each trial

Cycle number	Fe (μg/L)	Co (μg/L)	Mo (μg/L)
1	228	246	168
2	291	212	174
3	361	271	187
4	296	354	213
5	298	318	194

Fig. S1. Effect of different catalysts on degradation efficiency (Initial pH value of 3.7, catalyst concentration of 200 mg/L, tetracycline hydrochloride of 200 mg/L, PMS concentration of 400 mg/L, reaction time of 20 min).

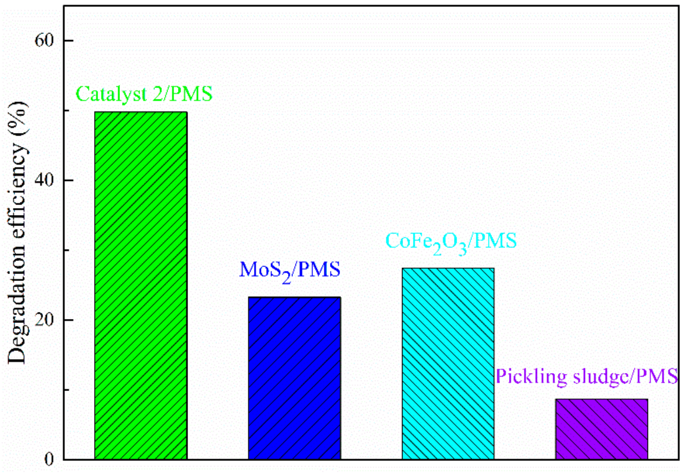

Fig. S2. EDX mapping image of the catalyst 2

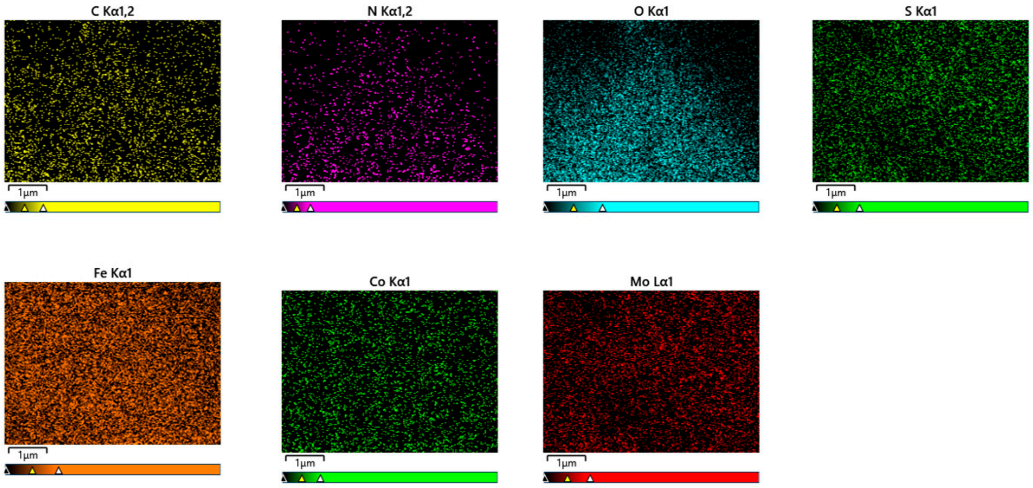

Fig. S3. EDS spectra of the catalyst 2

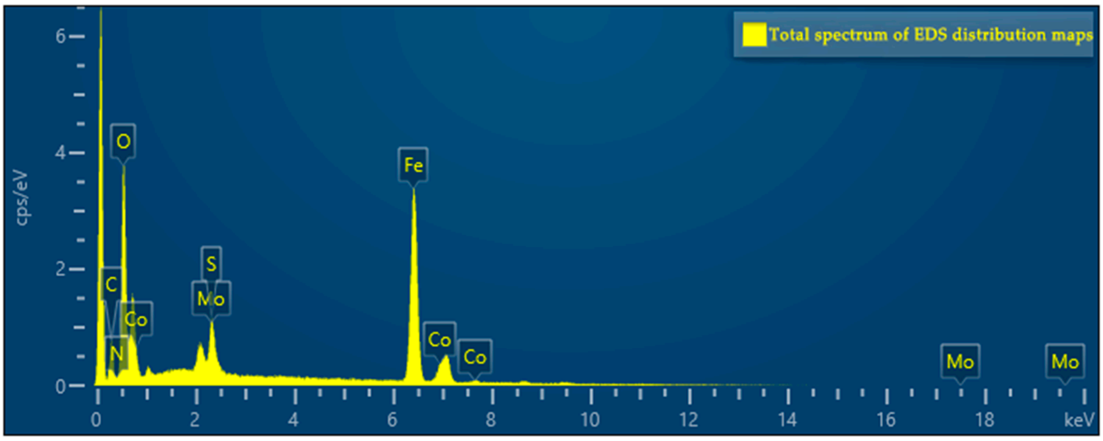

Fig. S4. XPS spectra of the prepared MoS_2: (a) S 2p spectrum in MoS_2; (b) Mo 3d spectrum in MoS_2.

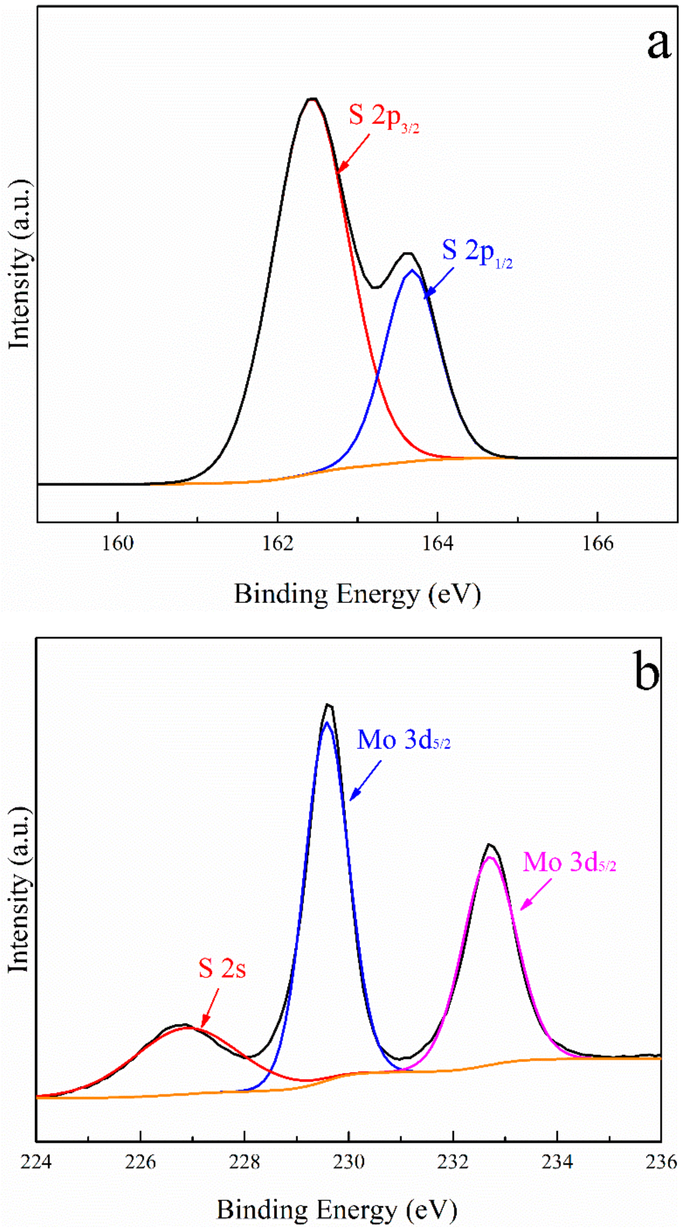

Fig. S5 COD removal of TCH solution during oxiadtion process

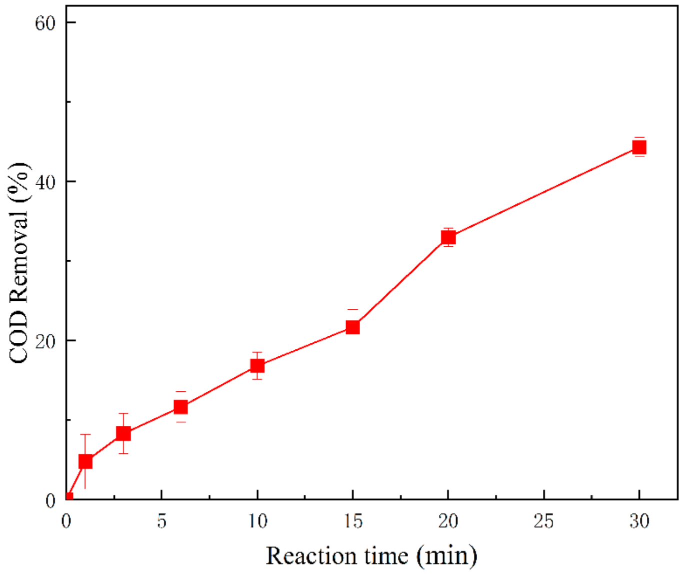

Fig. S6 Molecular structural formula of TCH.

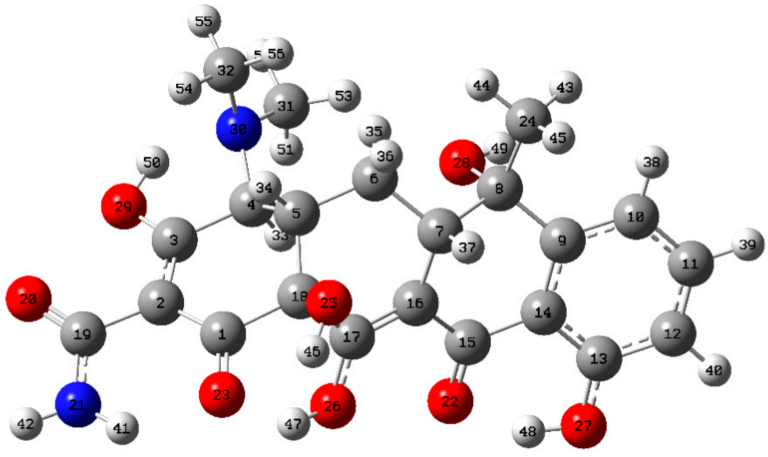

Fig. S7 SEM images of the $CoFe_2O_4$ layered catalyst after fifth cycle.

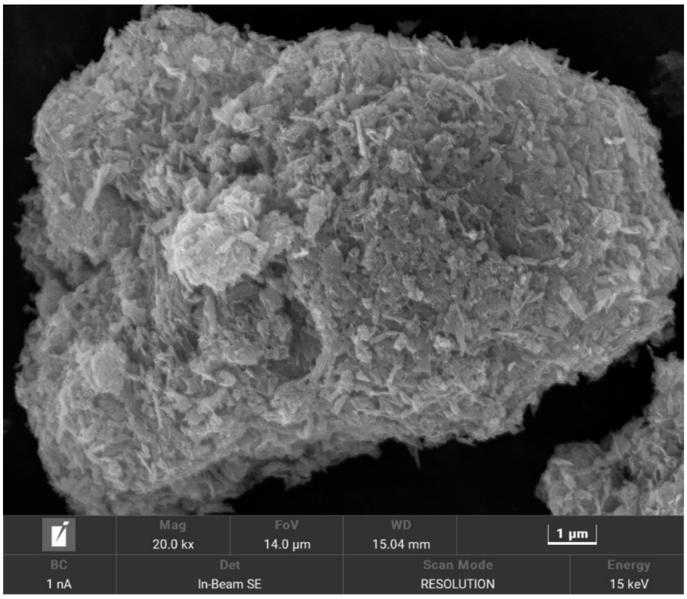

Supplement: Supplementary file 1 [file molecules-30-03194-s001.zip › molecules-3777488-supplementary.pdf]
